# Pattern of health care utilization and traditional and complementary medicine use among Ebola survivors in Sierra Leone

**DOI:** 10.1371/journal.pone.0223068

**Published:** 2019-09-27

**Authors:** Peter Bai James, Jon Wardle, Amie Steel, Jon Adams

**Affiliations:** 1 Australian Research Centre inw Complementary and Integrative Medicine, Faculty of Health, University of Technology Sydney, Ultimo, NSW, Sydney, Australia; 2 Faculty of Pharmaceutical Sciences, College of Medicine and Allied Health Sciences, University of Sierra Leone, Freetown, Sierra Leone; Sefako Makgatho Health Sciences University, SOUTH AFRICA

## Abstract

**Background:**

It is well established that Ebola Survivors experience a myriad of physical and psychological sequelae. However, little is known about how they seek care to address their health needs. Our study determines the current healthcare seeking behaviour among Ebola survivors and determines the prevalence, pattern of use and correlates of traditional and complementary medicine (T&CM) use among Ebola survivors in Sierra Leone.

**Methods:**

We conducted a nationwide questionnaire survey among a cross-sectional sample of Ebola Survivors in Sierra Leone between January and August 2018. We employed descriptive statistics, chi-square test, Fisher exact two-tailed test and backward stepwise binary regression analysis for data analysis. A p-value less than 0.05 was considered statistically significant.

**Results:**

Ebola Survivors who participated in our study (n = 358), visited a healthcare provider (n = 308, 86.0%), self-medicated with conventional medicines (n = 255, 71.2%) and visited a private pharmacy outlet (n = 141, 39.4%). Survivors also self-medicated with T&CM products (n = 107, 29.9%), concurrently self-medicated with conventional and T&CM products (n = 62, 17.3%), and visited a T&CM practitioner (n = 41, 11.5%). Almost half of (n = 163, 45.5%) Ebola survivors reported using T&CM treatments for post ebola related symptoms and non-Ebola related symptoms since their discharge from an Ebola treatment centre. Ebola survivors who considered their health to be fair or poor (AOR = 4.08; 95%CI: 2.22–7.50; p<0.01), presented with arthralgia (AOR = 2.52; 95%CI: 1.11–5.69, p = 0.026) and were discharged three years or less (AOR = 3.14; 95%CI: 1.13–8.73, p = 0.028) were more likely to use T&CM. Family (n = 101,62.0%) and friends (n = 38,23.3%) were the common sources of T&CM information. Abdominal pain (n = 49, 30.1%) followed by joint pain (n = 46, 28.2%) and back pain (n = 43, 26.4%) were the most cited post–Ebola indications for T&CM use. More than three-quarters of T&CM users (n = 135, 82.8%) failed to disclose their use of T&CM to their healthcare providers.

**Conclusion:**

Ebola survivors in Sierra Leone employ a myriad of healthcare options including T&CM in addressing their healthcare needs. Researchers, health policy makers and healthcare providers should be aware of the substantial role of T&CM in the health seeking of survivors, and this topic that should be factored into future research, policy formulation and implementation as well as routine practice regarding Ebola survivors.

## Introduction

Traditional and complementary medicine (T&CM) consists of numerous health products and practices (either originating within or outside of local native cultures) not traditionally connected with the conventional healthcare system and dominant medical profession.[1] Globally, interest in and use of T&CM either via self-medication or services provided by T&CM practitioners, has grown rapidly.[1] In Africa, a considerable number of the population use T&CM alone or in combination with conventional medicine to address their health needs even in instances where there is lack of scientific evidence to support the safety and efficacy of the therapy.[1, 2] The key reported drivers responsible for the high use of T&CM in Africa include perceived low relative cost; alignment with cultural, religious and spiritual values; and disenchantment with biomedicine.[2] T&CM use is considered a public health and health service issue as it presents opportunities and challenges for the provision of primary healthcare for many in low to mid-income countries.[1] In Sierra Leone, T&CM use is reported to be widespread especially among pregnant women[3], lactating mothers[4] hypertensive patients[5], infertile women[6] and for the treatment of malaria among children[7, 8] and adults.[9]

The increased incidence of emerging (e.g. Ebola, Zika and Severe acute respiratory synticial syndrome (SARS)) and re-emerging (malaria and tuberculosis) infectious diseases is linked to a range of biological, ecological, political and socioeconomic factors, and is considered a threat to global health security.[10, 11] In low-income countries, where health systems are often under-resourced and where medical pluralism (the use of both conventional and T&CM for health and illness) is rife[2, 12] emerging and re-emerging infectious diseases account for approximately half of all deaths.[13]. Due to widespread resistance to antimicrobials employed to treat emerging and re-emerging disease pathogens and associated safety concerns, there has been heightened public interest in T&CM to treat infectious diseases–supported by the perception that these therapies and treatments are natural and safe.[14] For instance, traditional Chinese medicine formulae that is made up of American ginseng Radix Adenophorae Strictae, Radix Ophiopognis, Radix Bupleuri, Radix Scutellariae, mulberry leaf, Cortex Lycii, Radicis, Herba Artemisiae, Rhizoma Phragmitis, Radix, Angelicae Sinensis, Radix Paeoniae Alba and Rhizoma Pinelliae have been used to treat patients with SARS[15]. While there are reports of T&CM use in SARS and Zika virus disease management,[16, 17] the extent and outcome of T&CM utilization among Ebola patients and survivors have received little or no research attention since the discovery of the disease.

The West African Ebola Virus disease (EVD) outbreak is described as the largest and most devastating health emergency the world has witnessed in the history of the EVD. As of 2016, the virus had infected over 28, 000 people and caused 11,310 deaths in the three most affected countries West African countries: Guinea, Liberia and Sierra Leone[18]. The Ebola outbreak in West Africa also recorded the maximum number of survivors of the disease to date. Over 7000 individuals in Guinea, Liberia and Sierra Leone were estimated to have survived EVD in 2016[19] and it is well established that in addition to the psychological trauma experienced during admission, most survivors present with immediate and long term physical and mental complications post-discharge[20–22]. Currently, there are fewer reports on the involvement of T&CM during Ebola outbreaks in Africa. These reports serve to highlight T&CM role in EVD transmission and prevention without offering a deeper insight into its pattern of use and or impact.[23–25] Preliminary unpublished data from Sierra Leone suggests EVD survivors experiencing long-term physical and psycho-social problems seek the services of traditional healers.[26] Such health seeking relating to T&CM may be due to personal, economic, psychological, social and cultural needs as well as health system factors. However, vigorous, large-scale studies are needed to determine the extent to which this initial report can be confirmed and representative of the wider population of Ebola survivors. In light of the common use of T&CM in Sierra Leone,[3, 5, 6] and the interest among the scientific community in understanding post-Ebola sequelae among survivors,[27] there is a significant gap in our understand of the role of T&CM in addressing the health needs of Ebola survivors (within the wider landscape of their health seeking behaviours). In direct response, the study reported here aims to determine the current healthcare seeking behaviour among Ebola survivors suffering from post-Ebola complications including an examination of the prevalence, pattern of use and correlates of T&CM use among Ebola survivors in Sierra Leone.

## Materials and methods

### Study setting, design, and participants

Sierra Leone is a low-income West African country with a population of 7million inhabitants and a land size of approximately 71,740 km^2^ [28].It borders Guinea in the northeast and Liberia in the Southeast. Sierra Leone is divided into four administrative regions (northern, southern, eastern provinces as well as the western area) and 14 districts. Despite substantial investment in the health sector, Sierra Leone still host to the worst health indicators, and the decline in the overall health status was excercebated by the 11years protracted civil war that ended in 2002, and the recent 2014–2016 Ebola outbreak. Whilst life expectancy at birth for males and females is currently at 52 years, maternal, infant and neonatal morbidity and mortality remain one of the highest in the world[29]. The health workforce is also limited with recent report estimating that there are 1.4 healthcare providers (doctor, nurses and midwives) per 10000 population[30].

**Fig 1:**
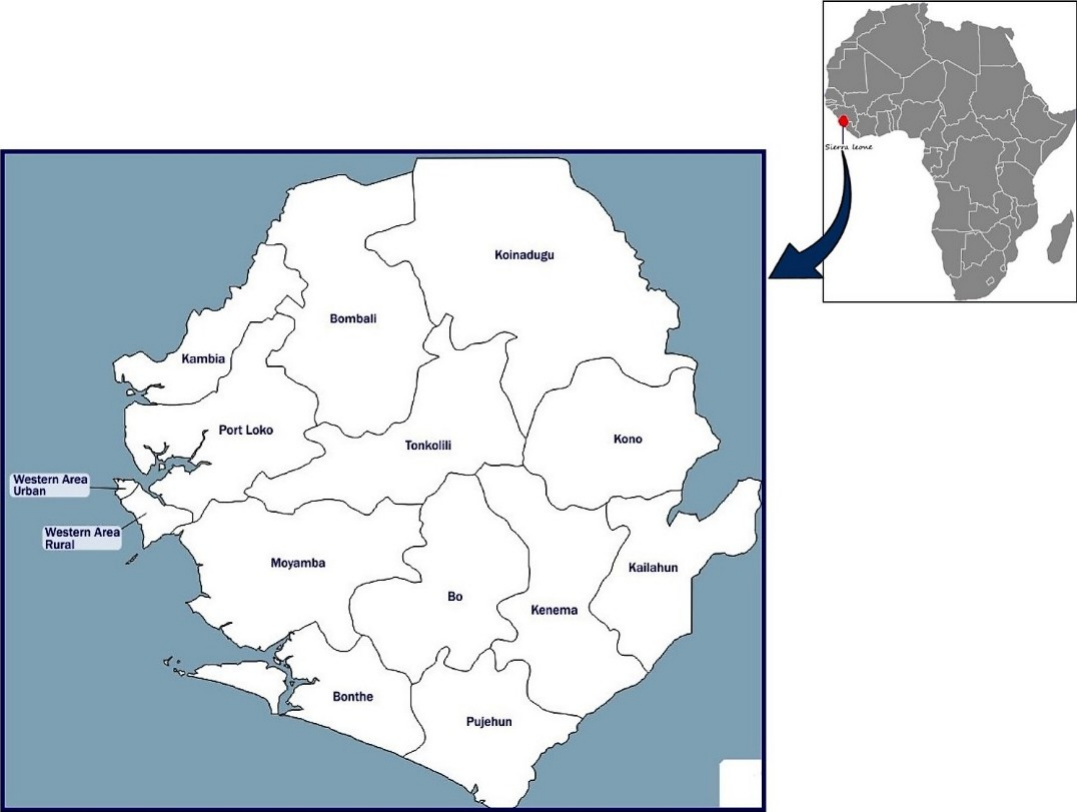
Locations of the five districts in Sierra Leone.

A cross-sectional nationwide study was conducted between January and August 2018 among Ebola survivors residing five districts across all four geographic regions (Western Area, Northern Province, Eastern Province and Southern Province) of Sierra Leone. **Fig 1**shows the locations of the five districts in Sierra Leone and they include western area urban and western area rural districts (both in the Western area), Bo district (Southern province), Kenema district (Eastern province) and Bombali district (Northern province). These districts were chosen based on the epidemiological profile of the total confirmed Ebola cases and because they are host to the highest number of Ebola survivors in Sierra Leone across all four geographic regions[31]. Male and female adult (at least 18years old) Ebola survivors with varied socio-demographic and health related characteristics experiencing post-Ebola sequelae were recruited into the study. Ebola Survivor status was confirmed by the presentation of their Ebola discharge certificates. Given that majority of Ebola survivors (76%-90%) of are experiencing post–Ebola symptoms [32, 33], determination of post-Ebola sequelae was based on EVD survivors’ self report of signs and or symptoms that occurred only after surviving the EVD. We excluded Ebola Survivors who presented with post-Ebola complications that seriously challenged their ability to accurately provide information or participate in the study. Examples include memory loss, hearing loss, high fever and bleeding or those experiencing acute emotional distress that will put the research team and study participants at risk. During recruitment, Ebola survivors were screened for acute emotional distress using a screening guide (**S1 File**) adapted from Draucker et al[34]. Ebola survivors were asked whether they are experiencing any form of emotional distress that is getting in the way of them doing their normal daily activities (school, work, family, and other obligations or whether they having thoughts of harming themselves or others.

### Sampling strategy

We determined the sample size based on the formula for sample size calculation for cross-sectional studies. We used a prevalence of 50% since no previous study has determined the prevalence of use of T&CM in this patient cohort. In this case, we assumed that 50% of the 4051[19] registered survivors in Sierra Leone would have used T&CM and with a confidence level of 95% and margin of error 5%, a minimum sample of 351 Ebola Survivors experiencing post-Ebola complications was calculated. To increase our statistical power, 400 survivors were invited to participate, but only 377 consented. Ebola survivors were sampled from five districts across all four geographical regions of Sierra Leone. For each district, the headquarter town was chosen as an urban settlement alongside a nearby rural setting which was chosen randomly from the rural settings located around the urban area. Simple random sampling was employed to recruit survivors in each of the five districts. The number of survivors targeted in each of the four regions was determined by proportional representation. EVD survivors were recruited via was done through telephone using the Sierra Leone Association of Ebola Survivors’ national database.

### Study questionnaire

A structured questionnaire (**S2 File)** was developed based on the reviewed literature.[2, 3, 20, 21] The final version of the questionnaire was informed by feedback from the pilot study conducted among 10 Ebola survivors whose data was not included in the final analysis and insights from a group of local experts (epidemiologist, pharmacologist, an infectious disease specialist and a physician providing care to Ebola survivors). The questionnaire had three domains. The first domain examined participant demographics and health related characteristics, the second focused upon health seeking behaviour, T&CM and products utilisation, information sources about T&CM and disclosure of T&CM to healthcare providers. The third domain explored Ebola survivors’ attitudes towards T&CM in which they were asked whether they agreed or disagreed with certain statements. Examples of the attitude statements include T&CM has fewer side effects than conventional medicine (CM), T&CM is more natural than CM and T&CM promotes a holistic approach to health. Four trained research assistants from the University of Sierra Leone were employed to help administer the questionnaire. After explaining the purpose of the study to all consented participants, data were collected via self-administered format for those that were literate or via face-to-face interviewer-administered format for those who were illiterate. Data collection took place at the Sierra Leone Association of Ebola Survivors regional offices or at EVD survivors’ homes where they were handed the questionnaires. Our conceptualisation of T&CM included biological based therapy (herbal medicine and animal extract), spiritual therapy (prayer/faith healing), alternative medicine systems (Chinese herbal medicine, and acupuncture), and physical therapy/body manipulations (massage therapy, traditional bone setting).

### Ethical consideration

We obtained ethical approval (see attached) for the study from the University of Technology Sydney Human Research Ethics Committee (UTS-HREC-ETH17-2080). We also obtained a reciprocal ethical clearance from the Sierra Leone Ethics and Scientific Review Committee (SLESRC). A written patient information and consent form explaining the purpose and scope of the study as well as the option of opting out if survivors felt uncomfortable at any time during the interview was provided to each recruited participant. For those who were illiterate, the purpose and scope of the study were explained in Creole (–widely spoken language in Sierra Leone) before obtaining their consent to participate. Signing and thumb printing (for illiterate participants) the consent form was taken as an expression of their willingness to participate. Verbal consent from community leaders/elders and written approval from local healthcare authorities were also sought prior to commencing the study.

### Data analysis

We used IBM SPSS Statistics version 24 to analyse our data. We recoded relevant independent variables to assist effective data analysis and interpretation. We used descriptive statistics to calculate frequencies and percentages for categorical variables and mean standard deviation for continuous independent variables. Chi-square and Fischer exact two-tailed tests were used to determine the associations between demographic variables (independent variables) and T&CM treatment use (dependent variable). We defined T&CM treatment use in our study as the use of the any of the T&CM modalities since discharge from Ebola treatment centre in at least one of the following scenarios, self-use of T&CM, concomitant self-use of T&CM and conventional medicines, visit to a religious leader/faith healer for healing and visit to traditional medicine practitioner (native doctor, herbalist). We employed backwards stepwise binary logistic regression to control for confounding and to establish the most parsimonious model that predicts the likelihood of T&CM treatment use. Significance level of the p-value was considered at 0.05.

## Results

Out of 377 EVD survivors who consented to participate in the study, 19 failed to complete the questionnaire resulting in 358 participants in the final dataset.

### Participant characteristics

Table 1 summarises EVD survivors’ demographic and health-related characteristics. The majority of participants were below the age of 42years (n = 276, 77.7%) and Muslim (n = 266, 74.3%). Approximately two-thirds were females (n = 223, 62.3%) and reported a monthly income of less than Le500, 000 (US$ 64.1). Arthralgia (n = 319, 89.1%) and headache (n = 272, 76.0%) were the common post-Ebola symptoms among EVD survivors.

**Table 1:** Socio demographic characteristics of EVD survivors (N = 358).

Characteristics	Variables	n (%)
Age group(years)	18–25	94(26.3)
	26–33	100(27.9)
	34–41	82(22.9)
	42–49	52(14.5)
	50–57	17(4.7)
	≥58	13(3.6)
Sex	male	135(37.7)
	Female	223(62.3)
Educational Status	Non-formal education	147(41.1)
	Primary	44(12.3)
	Secondary	126(35.2)
	Tertiary	41(11.5)
Religious Affiliation	Christianity	92(25.7)
	Islam	266(74.3)
Marital Status	Single	100(27.9)
	Married/Cohabitating	171(47.8)
	Divorced/Separated	4(1.1)
	Widow/widower	83(23.2)
Financial management	It is impossible/ Difficult all the time	110(30.7)
	difficult some time	238(66.5)
	Not too bad	10(2.8)
Monthly income (Leones)	Less than 500,000	252(70.4)
	500,000 - 1million	94(26.3)
	>1million	12(3.4)
Residential Area	Urban	219(61.2)
	Rural	139(38.8)
Region	Northern region (Bambali district)	120(33.5)
	Southern region (Bo district)	55(15.4)
	Eastern region (Kenema district)	62(17.3)
	Western Area	121(33.8)
Employment status before being infected with Ebola	Full time	230(64.2)
	Part time	19(5.3)
	Casual/Temporal	17(4.7)
	Looking for job	26(7.3)
	not in paid work force	66(18.4)
Employment status after surviving Ebola	Full time	164(45.8)
	Part time	16(4.5)
	Casual/Temporal	19(5.3)
	Looking for job	100(27.9)
	not in paid work force	59(16.5)
Current perceived health status	Very good	3(0.8)
	Good	93(26.0)
	Fair	190(53.1)
	Poor	72(20.1)
Duration(months) since discharged from ETC	Mean(standard deviation)	41.89(3.83)
	≤3 years	27(7.5)
	>3years	331(92.5)
Known Chronic disease	Yes	46(12.8)
	No	312(87.2)
Arthralgia(Joint pain)	Yes	319(89.1)
	No	39(10.9)
Headache	Yes	272(76.0)
	No	86(24.0)
Ocular Symptoms	Yes	206(57.5)
	No	152(42.5)
Fatigue	Yes	181(50.6)
	No	177(49.4)
Back pain	Yes	179(50.0)
	No	179(50.0)
Abdominal pain	Yes	132(36.9)
	No	226(63.1)
Auditory symptoms	Yes	61(17.0)
	No	297(83.0)
Skin Disorders	Yes	55(15.4)
	No	303(84.6)
Alopecia	Yes	38(10.6)
	No	320(89.4)

### Conventional and T&CM health service utilisation among EVD survivors

EVD survivors conventional and T&CM health-seeking behaviour since discharge from ETC are reported in Table 2; 308 survivors (86.0%) visited a healthcare provider (doctor, nurse at hospital, Ebola Survivor clinic etc.) while 255(71.2%) and self-medicated with conventional medicines and 141(39.4%) visited a private pharmacy outlet. Meanwhile, 107(29.9%), 62(17.3%) and 41(11.5%) self-medicated with T&CM product, self-used both conventional and T&CM concurrently and visited T&CM practitioner respectively. Close to three-quarters of EVD survivors who self-medicated with conventional medicines (191/255) and half of EVD survivors who self-medicated with T&CM product (54/107) or who concurrently self-medicated with both conventional and T&CM product, (31/62) reported doing so to treat their post-Ebola symptoms. Meanwhile, the majority of EVD survivors that visited a hospital/clinic (251/308), or visited a traditional medicine practitioner (30/41) did so to seek a complete cure for their Ebola sequelae. Slightly over half of EVD survivors that sought care at a private pharmacy (75/141) and close to two-thirds of EVD survivors who visited a faith/religious healer (47/73) did so to be completely cured from their post-Ebola symptoms.

**Table 2:** Prevalence of conventional and T&CM health service utilization among EVD survivors.

Conventional and T&CM health service utilization	Type of health care utilized	n (%)	Reasons for seeking particular health care model
	Self- medicated with Conventional medicine	255(71.2)	Managing my symptoms for my Ebola sequelae(191/255) Seeking a cure for my Ebola sequelae (94/255) Health concern unrelated to Ebola complication (12/255)
	Self-medicated with traditional and complementary medicine	107(29.9)	Managing my symptoms for my Ebola sequelae(54/107) Seeking a cure for my Ebola sequelae (32/107) Health concern unrelated to Ebola complication (37/107)
	Concurrent use of both conventional and traditional complementary medicine	62(17.3)	Managing my symptoms for my Ebola sequelae(31/62) Seeking a cure for my Ebola sequelae (28/62) Health concern unrelated to Ebola complication (4/62)
	Visited a healthcare provider (doctor, nurse at hospital, Ebola Survivor clinic etc.)	308(86.0)	Managing my symptoms for my Ebola sequelae (94/308) Seeking a cure for my Ebola sequelae (251/308) Health concern unrelated to Ebola complication (19/308)
	Visited a private pharmacy outlet	141(39.4)	Managing my symptoms for my Ebola sequelae (60/141) Seeking a cure for my Ebola sequelae (75/141) Health concern unrelated to Ebola complication (10/141)
	Visited a spiritual house (pastor/Imam in church/mosque)	73(20.4)	Managing my symptoms for my Ebola sequelae (20/73) Seeking a cure for my Ebola sequelae (47/73) Health concern unrelated to Ebola complication (6/73)
	Visited a traditional medicine practitioner (native doctor, herbalist)	41(11.5)	Managing my symptoms for my Ebola sequelae(4/41) Seeking a cure for my Ebola sequelae (30/41) Health concern unrelated to Ebola complication(9/41)

### Characteristics of T&CM users

The bivariate association between T&CM and demographic and health-related variables are reported in Table 3. Educational Status (p = 0.045), region (p<0.001), current perceived health status (p<0.001) and duration (months) since discharged from Ebola treatment centre (p = 0.022) show significant associations between T&CM users and non- T&CM users. The results of the backwards stepwise binary logistic regression are shown in Table 4 and it revealed that EVD survivors who considered their health to be fair or poor (AOR = 4.08; 95%CI: 2.22–7.50; p<0.01), presented with arthralgia (AOR = 2.52; 95%CI: 1.11–5.69, p = 0.026) and were discharged three years or less (AOR = 3.14; 95%CI: 1.13–8.73, p = 0.028) were more likely to use T&CM.

**Table 3:** Association between T&CM treatment use and sociodemographic variables.

Characteristics	Variables	Users n(%)	Non-users n(%)	p-value
Age group	18–33 years	91(55.8)	103(52.8)	0.760
	34–49 years	60(36.8)	74(37.9)	
	≥ 50 years	12(7.4)	18(9.2)	
Sex	Male	64(39.3)	71(36.4)	0.579
	female	99(60.7)	124(63.6)	
Educational Status	Non-formal education	63(38.7)	84(43.1)	0.045
	Primary	25(15.3)	19(9.7)	
	Secondary	63(38.7)	63(32.3)	
	Tertiary	12(7.4)	29(14.9)	
Religious Affiliation	Christianity	44(27.0)	48(24,6)	0.608
	Islam	119(73.0)	147(75.4)	
Marital Status	Single	45(27.6)	55(28.2)	0.258
	Married/Cohabitating	72(44.2)	99(50.8)	
	Divorced/Separated/widowed	46(28.2)	41(21.0)	
Financial management	It is impossible/ Difficult all the time	58(35.6)	52(26.7)	0.153
	difficult some time	100(61.3)	138(70.8)	
	Not too bad	5(3.1)	5(2.6)	
Monthly income (Leones)	Less than 500,000	117(71.8)	135(69.2)	0.773
	500,000 - 1million	40(24.5)	54(27.7)	
	>1million	6(3.7)	6(3.1)	
Residential Area	Urban	94(57.7)	125(64.1)	0.214
	Rural	69(42.3)	70(35.9)	
Region	Northern region (Bambali district)	33(20.2)	87(44.6)	<0.001
	Southern region (Bo district)	24(14.7)	31(15.9)	
	Eastern region (Kenema district)	21(12.9)	41(21.0)	
	Western Area	85(52.1)	36(18.5)	
Employment status after surviving Ebola	Full time	73(44.8)	91(46.7)	0.292
	Part time	8(4.9)	8(4.1)	
	Casual/Temporal	5(3.1)	14(7.2)	
	Looking for job	52(31.9)	48(24.6)	
	not in paid work force	25(15.3)	34(17.4)	
Current perceived health status	Very good/Good	23(14.1)	73(37.4)	<0.001
	Fair/poor	140(85.9)	122(62.6)	
Duration(months) since discharged from ETC	≤3 years	18(11.0)	9(4.6)	0.022
	>3years	145(89.0)	186(95.4)	
Known Chronic disease	Yes	19(11.7)	27(13.8)	0.538
	No	144(88.3)	168(86.2)	
Arthralgia	Yes	147(90.2)	172(88.2)	0.550
	No	16(9.8)	23(11.8)	
Headache	Yes	118(72.4)	154(79.0)	0.147
	No	45(27.6)	41(21.0)	
Ocular Symptoms	Yes	100(61.3)	106(54.4)	0.187
	No	63(38.7)	89(45.6)	
Fatigue	Yes	76(46.6)	105(53.8)	0.174
	No	87(53.4)	90(46.2)	
Back pain	Yes	85(52.1)	94(48.2)	0.458
	No	78(47.9)	101(51.8)	
Abdominal pain	Yes	65(39.9)	67(34.4)	0.281
	No	98(60.1)	128(65.6)	
Auditory symptoms	Yes	38(23.3)	23(11.8)	0.004
	No	125(76.7)	172(88.2)	
Skin Disorders	Yes	29(17.8)	26(13.3)	0.244
	No	134(82.2)	169(86.7)	
Alopecia	Yes	24(14.7)	14(7.2)	0.021
	No	139(85.3)	181(92.8)	

**Table 4:** Predictors of T&CM treatment use among EVD survivors using backwards stepwise binary logistic regression.

Characteristics	Variables	Adjusted OR^a^	95% CI^b^	p-value
Current perceived health status	Very good/Good	1		
	Fair/poor	4.08	2.22–7.50	**<0.001**
Region	Western Area	1		
	Northern region (Bambali district)	0.14	0.08–0.27	**<0.001**
	Southern region (Bo district)	0.37	0.18–0.78	**0.008**
	Eastern region (Kenema district)	0.19	0.09–0.40	**<0.001**
Duration(Years) since discharged from ETC	>3years	1		
	≤3 years	3.14	1.13–8.73	**0.028**
Arthralgia(Joint pain)	No	1		
	Yes	2.52	1.11–5.69	**0.026**
Headache	No	1		
	Yes	0.48	0.27–0.87	**0.014**
Abdominal Pain	No	1		
	Yes	1.57	0.94–2.62	0.084
Auditory symptoms	No	1		
	Yes	1.80	0.94–3.46	0.077
Fatigue	No	1		
	Yes	0.61	0.37–1.00	0.054

^a^OR = Odd ratio,

^b^ CI = Confidence interval

### Pattern of T&CM utilisation

Table 5 describes the pattern of T&CM treatment use among users. A total of 163 (45.5%) EVD survivors reported using a T&CM product and/or a T&CM practitioner since their discharge from the Ebola treatment centre. Biological-based therapy (n = 141, 86.5%) with was the most common T&CM treatment modality used among T&CM users. The most frequent reasons put forward by survivors for using T&CM include: the high cost of conventional medicine (n = 84, 51.5%); the alignment between T&CM and their own beliefs and faith (n = 84, 51.5%); and the perception that conventional medicine is not always effective, or was not at all effective, for their condition or the perception that T&CM is more effective than conventional medicine for their condition (n = 76, 47.2%). On the other hand, the main reasons for not using T&CM among non-users include their perceptions that T&CM is ineffective (n = 116, 59.5%) and advice from their healthcare provider not to use T&CM (n = 89,45.6%). EVD survivors mentioned other reasons (n = 63, 32.3%) for not using T&CM and these include not used to taking T&CM and T&CM is without standardised dose. Close to half of T&CM users (n = 75, 46.0%) were using T&CM irregularly while family (n = 101, 62.0%) followed by friends (n = 38, 23.3%) were identified as the main source of recommendations to use T&CM. Abdominal pain (n = 49, 30.1%) followed by arthralgia (n = 46, 28.2%) and back pain (n = 43, 26.4%) were the most cited post–Ebola indications for T&CM use while malaria (n = 32, 19.6%) was the most cited non-post-Ebola indications for T&CM use.

**Table 5:** Pattern of T&CM treatment use.

	Variables	n (%)
T&CM treatment use	Yes	163(45.5)
	No	195(54.5)
Type of T&CM treatment use n = 163- multiple choice	Biological based therapy	141(86.5)
	Herbal medicine	136 (83.4)
	Animal Extract	23(14.1)
	Prayer/faith healing	60(36.8)
	Acupuncture	1(0.61)
	Massage	33(20.2)
	Others (scarification, local surgery)	10(6.1)
Reasons for using T&CM treatment n = 163- multiple choice	Conventional medicine is not always effective/ was not effective for my condition/T&CM effective than conventional medicine for my condition	76(47.2)
	T&CM is quick or fast in action	53(32.5)
	I cannot afford to buy conventional medicine because it is too expensive	84(51.5)
	T&CM is natural	56(34.4)
	Conventional medicine has side effects	10(6.1)
	T&CM is more is more in keeping with one’s belief and faith	84(51.5)
	To promote and maintain one’s health	60(36.8)
	I want to have control over my health	18(11.0)
	Others	10(6.1)
Reasons for not using T&CM N = 195- multiple choice	I don’t believe it works	116(59.5)
	My healthcare provider advised not use T&CM	89(45.6)
	Caused side effects	56(28.7)
	Others	63(32.3)
Perceived adverse effect of T&CM treatment n = 163	Dizziness	15(9.2)
	Drowsiness	9(5.5)
	Itching	17(10.4)
	Nausea and vomiting	46(28.2)
	Others (headache, dysuria, weakness)	3(1.8)
	No side adverse effect	68(41.7)
Frequency of T&CM use n = 163	Irregular use	75(46.0)
	Daily	32(19.6)
	More than once per week	26(16.0)
	Weekly	30(18.4)
Recommenders of T&CM use n = 163- multiple choice	Family	101(62.0)
	Friends	38(23.3)
	Media(radio or TV)	5(3.1)
	Health care provider	5(3.1)
	Neighbour	17(10.4)
	Traditional medicine practitioner	33(20.2)
	Myself	14(8.6)
Post–Ebola Indications for T&CM use n = 163- multiple choice	Abdominal pain	49(30.1)
	Arthralgia	46(28.2)
	Back pain	43(26.4)
	Headache	32(19.6)
	Ocular problems	19(11.7)
	Auditory problems	8(4.9)
	Fatigue	23(14.1)
	Menstrual problems[Amenorrhea(7), heavy menstrual bleeding(2), irregular menstrual bleeding(4) ]	13(8.0)
	Skin disorders	9(5.5)
	Chest pain	2(1.2)
	Haemorrhoid	1(0.6)
	Infertility	1(0.6)
	Alopecia	1(0.6)
Non-post-Ebola indications for T&CM use n = 163- multiple choice	Malaria	32(19.6)
	Abdominal pain	8(4.9)
	Gynaecological problem (Menstrual pain(3), vaginal discharge and cleansing(2), STI(2))	7(4.3)
	Typhoid fever	3(1.8)
	Peptic ulcer	2(1.2)
	Pregnancy	2(1.2)
	Health maintenance	2(1.2)
	Infertility	1(0.6)
	Bone Fracture	1(0.6)
	Worm infection	1(0.6)
	Vomiting and diarrhoea	1(0.6)
	Constipation	1(0.6)
	Palpitation	1(0.6)
	Hemiplegia	1(0.6)

### Disclosure of T&CM use to healthcare providers

As shown in Table 6, more than three-quarters of T&CM users (n = 135, 82.8%) failed to disclose their use of T&CM to their healthcare providers. The main reason for non-disclosure was the fact that healthcare providers failed to ask about T&CM use during consultation (n = 77, 57.0%). Among those who disclosed their T&CM use status, the main reasons for disclosure were wanting healthcare providers to fully understand their health status (n = 15,53.6%) and being asked by healthcare providers about their T&CM use (n = 15,53.6%). Half of those who disclosed their T&CM use status considered their healthcare provider’s reaction to be discouraging (n = 14, 50.0%).

**Table 6:** Disclosure of T&CM use to healthcare providers.

Disclosure of TCAM use n = 163	Yes	28(17.2)
	No	135(82.8)
If YES, reasons for disclosure N = 28- multiple choice	I wanted them to fully understand my health status	15(53.6)
	I was concerned about drug interactions with the traditional and complementary medicine I was using	7(25.0)
	I thought they might know something about traditional and complementary medicine	3(10.7)
	They asked me about my use of traditional and complementary medicine use	15(53.6)
If YES, What was the reaction of the healthcare provider? N = 28	Encouraging	11(39.3)
	Discouraging	14(50.0)
	Neutral	3(10.7)
If NO, what were the reasons for non-disclosure N = 135- multiple choice	They did not ask me about my traditional and complementary medicine use	77(57.0)
	I did not think it was important	43(31.9)
	I did not think they would understand my choice	9(6.7)
	I was worried they would judge me or respond negatively/ will not provide the care I need	63(46.7)
	Traditional and complementary medicine is safe and so they did not need to know	5(3.7)
	There was not enough time in the consultation	7(5.2)
	I felt uncomfortable discussing it with them	6(4.4)
	I did not think they would know anything about traditional and complementary medicine	12(8.9)
	Others(My T&CM use is something personal)	1(0.7)

## Discussion

Our paper reports the findings of the first study (since the first Ebola outbreak more than 40 years ago) to examine the pattern of health care utilisation and T&CM use among Ebola survivors experiencing post-Ebola sequelae. The finding of this study suggests that Ebola survivors are seeking a myriad of healthcare options (conventional medicine and T&CM) and that medical pluralism is common in addressing their healthcare needs. Although the majority of survivors seek care from a conventional health provider, at the same time, we observed that a considerable number either self-medicate with either/or both conventional medicine and T&CM or seek care from a private pharmacy, religious leader (pastor/Imam in church/mosque) or from a T&CM practitioner. Although no comparable data regarding healthcare seeking behaviour among Ebola survivors exist in the literature, our findings are in line with the nature of health seeking behaviour reported in other emerging and re-emerging infectious diseases such as malaria among children in Sierra Leone,[7, 8], HIV/AIDS patients in South Africa[35] and tuberculosis patients in rural Nigeria.[36]. The use of multiple healthcare approaches may be a reflection of the high cost and the perceived lack of effectiveness of current allopathic treatments as well as the absence of specialised care for post–Ebola sequelae. For instance, our study revealed that EVD survivors’ perception of conventional medicine not being effective and unaffordable were the main reasons for survivors seeking T&CM treatment.

Our analyses shows that close to half of Ebola survivors had self-prescribed T&CM products and/or used the services of a T&CM practitioner since post–ETC discharge. In comparison with other emerging and re-emerging infectious diseases, our reported T&CM prevalence is similar to the average prevalence of T&CM product and practitioner use among HIV/AIDS patients in sub-Saharan Africa[2] but slightly lower than the frequency of T&CM use for the treatment of malaria among Sierra Leonean adults.[9] Also, the reasons cited in our study for using T&CM treatment are in line with previous studies on T&CM use in Sierra Leone[3, 5, 6, 9, 37] and across Africa,[2] where T&CM is reported to be highly accessible, affordable and culturally accepted. Within the Ebola virus disease context, the high use of T&CM among survivors may be explained in that current biomedical care as provided to survivors with post-Ebola complications is proving inadequate given that the nature and mechanism of these sequalae are still not well understood.[38] Also, the conventional health care services currently provided in West Africa are dominated by general health assessments and largely devoid of specialised care, especially for those survivors experiencing severe and long term sequalae[27].

The widespread use of T&CM together with conventional medicine among EVD survivors in our study indicates that the Sierra Leone free healthcare initiative (FHCI) has not been effective in preventing EVD survivors from using traditional and complementary health approaches. The FHCI policy was originally designed for pregnant women, lactating mothers and under-five children[39] and EVD survivors in Sierra Leone have recently being included as beneficiaries.[40] Interestingly, recent Sierra Leonean studies indicate that T&CM use is common among pregnant women and lactating mothers seeking care in public healthcare facilities [3, 4]. This shows that the high cost of treatment that impedes access to biomedical care is not the only driver for T&CM use among EVD survivors including pregnant women and lactating mothers but a combination of social, cultural and health system factors may play a role. Also, given that individuals with chronic complex diseases are seeking more holistic/person-centred care [41, 42], it may be that survivors are seeking other health services if the available health care is not meeting these other needs. Although our study reports some of the reasons for T&CM use among EVD survivors, a qualitative study is needed to offer a deeper understanding of the reasons why EVD survivors still seek traditional and complementary healthcare even when medical care is free. Given T&CM is highly perceived as effective and in line with people’s cultural and religious values[2], the use of T&CM among survivors who seek conventional care in our study also indicates the need for an open discussion between T&CM users and healthcare providers about the benefits and risks of T&CM so that survivors can make an informed decision.

The high use of T&CM and the pluralistic health seeking behaviour among Ebola survivors may present challenges for post-Ebola care in Sierra Leone. Such challenges include adverse effects resulting from herbal-drug interactions and compromised safety due to herbal medicine contamination can negatively affect the outcome of care received by Ebola survivors. Also, the fact that our study reports that most survivors use T&CM irregularly and, that the decision to use T&CM was influenced by families or friends raises concern with regards the effective, judicious use of T&CM among Ebola survivors. Much information on T&CM from such non-professional sources may well be incorrect and lacking an evidence-base and, as such, may potentially undermine patient health by enhancing therapeutic failure of conventional medicine,[43] conventional medicine toxicity[44] and non-adherence to pharmaceuticals [45]. Thus, the potential for direct and indirect risks associated with medical pluralism emphasizes the need to educate Ebola survivors on the risks and benefits of commonly used T&CM in Sierra Leone.

As reported in previous T&CM use studies in Sierra Leone[3, 5, 6] and across Africa[2], the fact that most survivors in our study failed to disclosed their T&CM use status to their healthcare provider is a cause for concern. Such non-disclosure highlights largely, the absence of communication between healthcare providers and Ebola survivors regarding T&CM. Fear of being judged or not receiving care as well as lack of inquiry from healthcare providers were cited as the main reasons for non-disclosure amongst our study participants, and this mirrors findings of studies conducted among general and sub-health populations across Africa.[2] Given that there is an established relationship between non-disclosure of T&CM use and patient safety,[46] educational interventions targeting healthcare practitioners providing care to survivors will be useful in attracting attention and improving knowledge about T&CM use in the care for Ebola survivors[47]. Even in cases, where survivors disclose their T&CM use, our finding indicates that healthcare providers’ response was discouraging mirroring findings from a study conducted among HIV/AIDS patients in South Africa[48]. This speaks to the fact that the nature of communication between healthcare providers and Ebola survivors was often devoid of respect and empathy. Effective communication around T&CM use with Ebola survivors is essential to optimizing their care. Thus, it is important that healthcare providers be mindful that Ebola survivors are potential T&CM users and be proactive in initiating respectful conversations around survivors’ T&CM use.

Regression analyses of our data show that Ebola survivors who consider their health to be poor/fair were more likely to use T&CM. The correlation between perceived poor health of EVD survivors and their use of T&CM use is in line with results from a study of T&CM use among the general population in 32 countries around the world.[49] The relationship between EVD survivors’ perceived poor health and their use of T&CM may be due to Ebola survivors’ unmet needs. For example, Ebola survivors in our study cited the ineffectiveness of conventional therapies to treat post-Ebola sequelae as one of the reasons for using T&CM in our study. The need for holistic and person-centred care as it has been reported among patients with chronic conditions [41, 50], may also explain why survivors who perceived their health to be poor are likely T&CM users. Ebola survivors who discharged from an Ebola treatment centre (ETC) three years or less to the time of data collection compared to those discharged more than three years prior to data collection were more likely to use T&CM. Such a relationship may be explained by the survivors’ increased utilization of various healthcare options particularly T&CM[51] given that survivors who were recently discharged from ETC are known to experience an increased number of severe post–Ebola sequalae[52]. Analyses of our data also indicated that EVD survivors who presented with arthralgia (joint pain) were more likely to use T&CM. It is possible that EVD survivors may decide to try alternate health approaches when the joint pain becomes recurrent or persistent after using conventional medicine. Further research is required to explore the reasons why survivors who perceive their health to be poor/fair, discharged from ETC three years or less and presented with arthralgia are likely to be T&CM users. Nevertheless, our findings have indicated that perceived health status, post-ETC discharge time and arthralgia are potential predictors for T&CM use among Ebola survivors and, that healthcare providers should take note of such details when identifying potential T&CM users among Ebola survivors.

Our study reports musculoskeletal problems (abdominal pain, arthralgia and back pain) as the most common post–Ebola sequalae among survivors who use T&CM. Such a finding is not surprising given that musculoskeletal problems have been reported to be common among Ebola survivors in Sierra Leone[53, 54]and that T&CM use is widespread in people with chronic musculoskeletal disorders[55]. As reported in our study, reasons cited in the literature to justify the use of T&CM for musculoskeletal problems include its perceived effectiveness, low cost, accessibility, alignment with patient’s cultural and spiritual beliefs of illness and health and dissatisfaction with conventional healthcare[41]. Our analyses also indicate that malaria was the most common non-post-Ebola indication for T&CM use among Ebola survivors. Malaria is a public health priority in Sierra Leone due to its high burden among the general population and especially among children[56]. Increased healthcare utilization due to malaria have been reported[56], and the use of T&CM which has been reported in the general Sierra Leonean population is certainly one of the myriad of healthcare approaches used to treat malaria[7–9]. The use of T&CM for post–Ebola musculoskeletal problems and malaria among survivors have significant implications for policy and practice with regards to the need for inclusion of T&CM as part of routine clinical consultation and the need for appropriate T&CM regulation.

## Limitations

Our study findings should be interpreted with the following limitations in mind. First, the data collected was based on self-report, which is a risk factor to responder and recall bias. Also, no cause-effect relationship can be inferred because of the cross-sectional design that we employed in this study. In addition, our study did not provide any information on survivors’ health outcomes due to T&CM use in particular. We excluded survivors who presented with serious post-ebola complications such as memory loss, hearing loss or acute severe emotional distress. However, this is the first nationwide survey that looks at healthcare utilization among Ebola survivors with an emphasis on their T&CM use and, therefore, our findings are representative of the wider population of Ebola survivors of Sierra Leone. In addition, our study will serve as a basis for future research in this area and other emerging and re-emerging infectious diseases outbreaks.

## Conclusion

Ebola survivors in Sierra Leone utilise a variety of health approaches including T&CM to address their post-discharge healthcare challenges. Thus, it is essential that researchers, health policymakers and healthcare providers consider T&CM in their research, policy formulation and implementation and routine practice with regards Ebola survivors.

## Supporting information

S1 File Screening interview guide for emotional distress. (DOCX)

S2 File Study questionnaire: Health care utilization, and traditional and complementary medicine (T&CM) use among Ebola survivors in Sierra Leone. (DOCX)
