# Supplementary material for: Pattern of health care utilization and traditional and complementary medicine use among Ebola survivors in Sierra Leone
Source: PLoS One. 2019 Sep 27;14(9):e0223068. doi: 10.1371/journal.pone.0223068 (PMC6764668; doi:10.1371/journal.pone.0223068)
Supplement: S1 File — (DOCX) [file pone.0223068.s001.docx]

TRADITIONAL AND COMPLEMENTARY MEDICINE USE AMONG EBOLA SURVIVORS STUDY

**SCREENING INTERVIEW GUIDE**

An experienced mental health professional and I will conduct all screening

This is Peter James and Dr X (mental health practitioner) from the University of Technology Sydney and University of Sierra Leone. We are contacting you for possible recruitment into our study that looks at traditional and complementary medicine use among Ebola survivors.

Do you have any question about the study? (If yes, answer question(s). If no, proceed)

Could you please verify your name, address and phone number?

Because this topic of traditional and complementary medicine use among Ebola survivors can be sensitive and might bring tough feelings, we are advising individuals who are experiencing high level of stress or emotional distress or whose participation might put them in danger not to participate at this time. Is it alright, if we ask you some questions to determine if there is any reason(s) you should not participate? (If NO, thanks for your time and interest. If **YES**, proceed to conduct a screening interview)

| **Screening Questions** | **YES** | **NO** | **Follow-up Questions**  **If YES, ask questions** | **Caller Response** | Acute Emotional distress or safety concern  (Y or N) | Imminent Danger  (Y or N) |
| --- | --- | --- | --- | --- | --- | --- |
| Are you experiencing a high level of stress or any emotional distress? |  |  | 1. Tell me what you are experiencing?  2. Is it getting in the way of you doing things you need to do (school, work, family, other obligations?  3. Is getting in way of you taking care of yourself?  4. Have been in the hospital recently for this problem? |  |  |  |
| Are currently having thoughts of harming yourself? |  |  | 1. Tell me what thoughts you are having?  2. Do you intend to harm yourself?  3. How do you intend to harm yourself?  4.Have been in the hospital recently for this problem |  |  |  |
| Are currently having thoughts of harming someone else |  |  | 1. Tell me what thoughts you are having?  2. Who do you intend to harm?  3. How do you intend to harm the him/her/them?  4. When do you intend to harm him/her/them?  5. Do you have the means to harm him/her/them? |  |  |  |
| If you participate in this study, would you be in any danger if anyone else found out |  |  | 1. How might you be in danger?  2. How might the other person find out you are participating?  3. What do you think the other person will do if they found out you are participating in this study? |  |  |  |

**Actions for screeners**

1. If answers to screening questions are all NO, read the confidential statement below and schedule an interview

CONFIDENTIALITY STATEMENT

*All answers that you give will be kept private. This because our study adheres to the national and international ethics requirement. This means anything you tell us will not be share with anyone unless it is required by law or you authorised us to do so.*

1. If the participant response reflect acute distress or safety concern but NOT an imminent danger, take the following steps
2. Do not schedule an interview
3. Recommend that the person contact a mental health practitioner at any of the public hospitals or our mental health practitioner for our study for follow-up
4. Indicate that with the permission of the individual our mental health practitioner for our study will call him/her the next day to see if he/she is okay
5. If a participant responses to additional questions reflect an imminent danger
6. Contact local law authorities
7. Indicate that, with the participant permission, our mental health practitioner of the study will contact him/her the next day to see if he/she is okay
