# Supplementary material for: Pattern of health care utilization and traditional and complementary medicine use among Ebola survivors in Sierra Leone
Source: PLoS One. 2019 Sep 27;14(9):e0223068. doi: 10.1371/journal.pone.0223068 (PMC6764668; doi:10.1371/journal.pone.0223068)
Supplement: S2 File — (DOCX) [file pone.0223068.s002.docx]

**Questionnaire Survey**

**ID Number……………………**

**Pattern of Health care utilization, and Traditional, and Complementary Medicine** (**T&CM) use among Ebola Survivors in Sierra Leone**

Please read this introduction carefully, as it will help you answer the questions.

Thank you for taking the time to complete this questionnaire. The questionnaire is designed to examine use of complementary/alternative health therapiesuse among Ebola survivors in Sierra Leone.

Traditional and complementary medicine (T&CM) are a group of either indigenous or imported health practices and system that are outside the mainstream healthcare system such as any natural health products (example, traditional herbal medicine/products, Chinese medicine product) or therapies (example, acupuncture, Massage, prayer/spirituality, homeopathy, yoga, meditation)

**Part A seeks information about you and your general health status.**

**Part B seeks information about your health seeking behaviour and pattern of T&CM use among Ebola Survivors**

**PART C accesses Attitudes towards traditional & complementary medicine**

**Part A**

## 1) What age group do you belong? Please select the response that best applies to you

| 18-25 |  |
| --- | --- |
| 26 – 33 |  |
| 34 – 41 |  |
| 42 – 49 |  |
| 50 – 57 |  |
| 58 – 65 |  |
| Above 65 |  |

## 2) What is your gender? Please select the response that best applies to you

    ○ Male       ○ Female

## 3) What highest educational level have you completed? Please select the response that best applies to you

| Non-formal education | ○ |
| --- | --- |
| Primary | ○ |
| Secondary | ○ |
| Tertiary education (University/Polytechnic/College of education) | ○ |

## 4) What is your Religious Affiliation? Please select the response that best applies to you

| Traditional religion | ○ |
| --- | --- |
| Christian Catholic | ○ |
| Christian Pentecostal | ○ |
| Christian Anglican | ○ |
| Muslim | ○ |
| Others (specify)…………….. | ○ |
|  |  |

## 5) What is your marital status? Please select the response that best applies to you

| Single | ○ |
| --- | --- |
| Married/living with a partner | ○ |
| Divorce/separated | ○ |
| Widowed | ○ |

| 6) What best describes your employment status beforebeing infected with Ebola? (*Mark one only*) | |
| --- | --- |
| Full-time work (35 hours per week) | ○ |
| Part-time work (15-30 hours a week) | ○ |
| Casual/temp work (irregular hours) | ○ |
| Looking for work | ○ |
| Not in the paid workforce | ○ |
| Other (please specify)________________ | ○ |

| 7)What best describes your CURRENT employment status (Mark one only) | |
| --- | --- |
| Full time work (35 hours per week) | ○ |
| Part time work (15-30 hours a week) | ○ |
| Casual/temp work (irregular hours) | ○ |
| Looking for work | ○ |
| Not in the paid workforce | ○ |
| Other (please specify)________________ | ○ |

| 8) How do you manage financially at the moment? (*Mark one only*) | |
| --- | --- |
| It is impossible | ○ |
| It is difficult all of the time | ○ |
| It is difficult some of the time | ○ |
| It is not too bad | ○ |
| It is easy | ○ |

| 9) What is your household Income per month (Leones)? (*Mark one only*) | |
| --- | --- |
| <500,000 | ○ |
| 500,000-1million | ○ |
| 1,001,000 - 2,000,000 | ○ |
| 2,001,000 – 3,000,000 | ○ |
| 3,001,000 – 4,000,000 | ○ |

| 10) What region of the country do you reside (*Mark one only*) | |
| --- | --- |
| North | ○ |
| South | ○ |
| East | ○ |
| Western Area | ○ |

## 11) Residence

Urban ○ Rural ○

12) Please indicate below the number of months since youwere discharged from the Ebola Treatment centre…………………………………………………………………………………

| 13) WHAT were the sign and symptoms you presented WITH WHENyouwere admitted at the Ebola Treatment CENTRE (MARK all that apply) | |
| --- | --- |
| Fever | ○ |
| Diarrhoea | ○ |
| Eye redness | ○ |
| Headache | ○ |
| Nausea and vomiting | ○ |
| Fatigue | ○ |
| Others (please specify) | ○ |

## 14) How long did you spend in the Ebola Treatment centre (days)? …………………….

| 15) How best can you describe the current status of your health? (*Mark one only*) | |
| --- | --- |
| Excellent | ○ |
| Very good | ○ |
| Good | ○ |
| Fair | ○ |
| Poor | ○ |

| 16) What type of post –Ebola complications are you currently suffering from? Please tick all that apply | |
| --- | --- |
| Arthralgia( pain in the joints) | ○ |
| skin disorders(skin problems) | ○ |
| Auditory symptoms (Ear problem) | ○ |
| Ocular symptoms(Eye problem) | ○ |
| headache | ○ |
| alopecia (loss of hair on your head | ○ |
| Back pain(pain in your back) | ○ |
| abdominal pain(pain in and around your stomach area | ○ |
| Fatigue(feeling tired) | ○ |
| Others (please specify) | ○ |

## 17)Did youhave any known CHRONIC DISEASEBEFORE GETTINGEbola?

Yes○ No ○

| 17a) If Yes, please tick the type of disease(tick all that APPLIES) | |
| --- | --- |
| Hypertension(High blood pressure) | ○ |
| Diabetes(high blood sugar) | ○ |
| Peptic ulcer (stomach ulcer- a break or an erosion of the lining of the stomach ) | ○ |
| Pulmonary disease(lung problem) | ○ |
| Rheumatoid arthritis (pain and swelling of the joints)) | ○ |
| Others (please specify) | ○ |

## 18) Please provide the names of western medicine you are currently taking or have taken for yourpost-ebola complication.

………………………………………………………………………………………………….. ……………………………………………………………………………………

………………………………………………………………………………………………….. ……………………………………………………………………………………

………………………………………………………………………………………………….. ……………………………………………………………………………………

**Part B: Health seeking behaviour and pattern of T&CM use of Ebola Survivors**

| 19) Since your discharge from ETU, how usually have you accessed healthcare for your condition? Please select the responses that best apply to you | | | | | | |
| --- | --- | --- | --- | --- | --- | --- |
|  | |  | Managing my symptomsfor my Ebola sequelae | Seeking a cure for my Ebola sequelae | Health concern unrelated to Ebola complication | Others |
| a) Self- medicated with western medicine only |  |  | ○ | ○ | ○ | ○ |
| b) Self-medicated with traditional and complementary medicine only |  |  | ○ | ○ | ○ | ○ |
| c)Self-medicated with both western and traditional medicine |  |  | ○ | ○ | ○ | ○ |
| d)Visited a healthcare provider (doctor, nurse at hospital, Ebola Survivor clinic etc.) only |  |  | ○ | ○ | ○ | ○ |
| e) Visited a pharmacy outlet |  |  | ○ | ○ | ○ | ○ |
| f) Visited a pastor/church/ Imam /mosque |  |  | ○ | ○ | ○ | ○ |
| g) Visited a traditional medicine practitioner (native doctor, herbalist) only |  |  | ○ | ○ | ○ | ○ |

**If you tick the options 19b, c, f& gabove Please answer the following questions (20A-20H) below and go to part C. If you tick options 19a,d&e please go straight to the next section i.e. Part C**

## 20a) Which of the following traditional and complementary medicines/practices (T&CM)have youused in the past twelve months?TICK ALL THAT APPLIES

**Biological products**:

Herbal medicines ○ Animal Extracts ○

Others specify ……………………….

**Spiritual Therapy:**

Faith/prayer healing ○

others specify ……………………….

**Alternative Medicine Systems:**

Chinese medicine ○ Acupuncture ○

Others specify ……………………….

**Physical Therapy/body Manipulation**:

Massage ○ others specify ………

**Others:**

Local Surgery/scarification ○ Ritual sacrifice ○

Urine therapy ○others specify ………

## b) what are your reasons for using TRADITIONAL AND COMPLEMENTARY MEDICINES/practicesyou ticked in 20a above?

Please **tick** all that as applies to you

1) Western medicine is not always effective [ ]

2) Traditional and complementary medicines is quick or fast in action [ ]

3) Western medicine is too expensive [ ]

4) Traditional and complementary medicines is natural [ ]

5) Western medicine has side effects [ ]

6)Traditional and complementary medicines is more in keeping with one’s belief and faith [ ]

7) To promote and maintain one’s health [ ]

8) I want to have more control over my health

9) Others (please specify) …………………

## C)Did you used any of the TRADITIONAL AND COMPLEMENTARY MEDICINES/PRACTICES YOU TICKED IN 20A ABOVE, for your post-ebola complication?

Yes [ ]

No [ ] Pleasespecify here what it was used for………………………………………………………………………………………………………..

If you tick “Yes”to 20C above, please answer questions 20D to 20H. If you tick “No” to 20C above, please answerquestions 20E-20H

| D) If it was for post-Ebola complications which of the following indication(s) did you use it for? (Tick all that apply) | |
| --- | --- |
| Arthralgia(pain and selling of the joints) | ○ |
| Fatigue(feeling of being tired) | ○ |
| abdominal pain(pain in and around the stomach area) | ○ |
| headache | ○ |
| skin disorders(skin problems) | ○ |
| Back pain | ○ |
| Alopecia(loss of hair in the head) | ○ |
| Auditory symptoms ( Hearing problem) | ○ |
| Ocular symptoms(eye problem) | ○ |
| Others (specify)…………………………………….. | ○ |

| E) how often do you use TRADITIONAL AND COMPLEMENTARY MEDICINES products/ practice you ticked in 20a? | |
| --- | --- |
| Irregular use | ○ |
| Daily | ○ |
| More than once per week | ○ |
| Weekly | ○ |

| F) WHO recommeNded the use OF TRADITIONAL AND COMPLEMENTARY MEDICINESYOU TICKED IN 20A? ?(Tick all that apply) | |
| --- | --- |
| Family members | ○ |
| Friends | ○ |
| Media(radio TV and internet) | ○ |
| Health practitioner | ○ |
| Neighbours | ○ |
| Traditional medicine practitioner | ○ |

**We want to understand the nature of communication with your healthcare providerconcerning your use of traditional and complementary medicine. Please answer the following**

| G) Did you tell your doctor, nurse or pharmacist about traditional and complementary medicine you are using? |  |
| --- | --- |
| Yes, I told them about all traditional and complementary medicines I was using | ○ |
| Yes, but I only told them about some of my traditional and complementary medicine use | ○ |
| No, I did not tell them about my traditional and complementary medicine use | ○ |
| If YES, what were the reasons for disclosing the use of traditional and complementary medicine to your doctor, nurse or pharmacist? |  |
| I wanted them to fully understand my health status | ○ |
| I was concerned about drug interactions with the traditional and complementary medicine I was using | ○ |
| I thought they might know something about traditional and complementary medicine | ○ |
| They asked me about my use of traditional and complementary medicine | ○ |
| If YES what was the reaction of the healthcare provider? |  |
|  |  |
| Encouraging | ○ |
| discouraging | ○ |
| Neutral | ○ |
| If NO, what were the reasons for not disclosing the use of traditional and complementary medicine to your doctor, nurse or pharmacist? |  |
| They did not ask me about mytraditional and complementary medicine use | ○ |
| I did not think it was important | ○ |
| I did not think they would understand my choice | ○ |
| I was worried they would judge me or respond negatively | ○ |
| Traditional and complementary is safe and so they did not need to know | ○ |
| There was not enough time in the consultation | ○ |
| I felt uncomfortable discussing it with them | ○ |
| I did not think they would know anything about traditional and complementary medicine | ○ |
| Others Please specify……………………………………………………………………………………… |  |

| h) If you did not use TCM, what was the reason? Tick all that apply) | |
| --- | --- |
| I don’t believe it works | ○ |
| My healthcare provider advised not use TCM | ○ |
| Caused side effects | ○ |
| Others (specify………………………………………….. | ○ |

**PART C. Attitudes towards traditional & complementary medicine**

## How much do you agree or disagree with the following statements? Please select the response per row that best applies to you.(Please tick only one option in each row)

|  |  | Strongly agree | Agree | Neutral | Disagree | Strongly Disagree | Don’t know |
| --- | --- | --- | --- | --- | --- | --- | --- |
| a | Traditional & complementary medicine is more natural than conventional medicine | ○ | ○ | ○ | ○ | ○ | ○ |
| b | Traditional & complementary medicine boosts my immune system/resistance | ○ | ○ | ○ | ○ | ○ | ○ |
| c | Traditional & complementary medicine has fewer side effects than conventional medicine | ○ | ○ | ○ | ○ | ○ | ○ |
| d | Traditional & complementary medicine promotes a holistic approach to health | ○ | ○ | ○ | ○ | ○ | ○ |
| e | Traditional & complementary medicine gives me more control over my health/body | ○ | ○ | ○ | ○ | ○ | ○ |
| f | Knowledge about the evidence of traditional & complementary medicine is important to me as a patient | ○ | ○ | ○ | ○ | ○ | ○ |
| g | My personal experience of the effectiveness of traditional & complementary medicine is more important than clinical evidence | ○ | ○ | ○ | ○ | ○ | ○ |
| h | Traditional & complementary medicine is a better preventive measure than conventional medicine | ○ | ○ | ○ | ○ | ○ | ○ |
| i | Traditional & complementary medicine needs to be tested for safety/side effects | ○ | ○ | ○ | ○ | ○ | ○ |

## How much do you agree or disagree with the following statements? Please select the response per row that best applies to you.

|  |  | Strongly agree | Agree | Neutral | Disagree | Strongly Disagree | Don’t know |
| --- | --- | --- | --- | --- | --- | --- | --- |
| a | Atraditional & complementary health practitioner spends a longer time with patients in consultations compared with a medical doctor | ○ | ○ | ○ | ○ | ○ | ○ |
|  |  |  |  |  |  |  |  |
| b | Atraditional & complementary health practitioner provides more support to his/her patient than a medical doctor does | ○ | ○ | ○ | ○ | ○ | ○ |
|  |  |  |  |  |  |  |  |
| c | I find it easier to talk to an traditional& complementary health practitioner that to a medical doctor | ○ | ○ | ○ | ○ | ○ | ○ |
|  |  |  |  |  |  |  |  |
|  |  |  |  |  |  |  |  |
|  |  |  |  |  |  |  |  |
| d | Medical doctors should be able to advise their patients about commonly used traditional & complementary medicines | ○ | ○ | ○ | ○ | ○ | ○ |
| e | My preferred care options were respected and supported by mymedical doctor | ○ | ○ | ○ | ○ | ○ | ○ |
|  |  |  |  |  |  |  |  |
| f | It is important to me that my preferred care options are respected and supported by myhealth care team | ○ | ○ | ○ | ○ | ○ | ○ |

***Thank You for your time***
